# *Lactobacillus paracasei* CCFM1229 and *Lactobacillus rhamnosus* CCFM1228 Alleviated Depression- and Anxiety-Related Symptoms of Chronic Stress-Induced Depression in Mice by Regulating Xanthine Oxidase Activity in the Brain

**DOI:** 10.3390/nu14061294

**Published:** 2022-03-18

**Authors:** Mengshu Xu, Peijun Tian, Huiyue Zhu, Renying Zou, Jianxin Zhao, Hao Zhang, Gang Wang, Wei Chen

**Affiliations:** 1 State Key Laboratory of Food Science and Technology, Jiangnan University, Wuxi 214122, China; 6190112118@stu.jiangnan.edu.cn (M.X.); 7160112069@vip.jiangnan.edu.cn (P.T.); 6200112124@stu.jiangnan.edu.cn (H.Z.); 6180112134@stu.jiangnan.edu.cn (R.Z.); zhaojianxin@jiangnan.edu.cn (J.Z.); zhanghao61@jiangnan.edu.cn (H.Z.); chenwei66@jiangnan.edu.cn (W.C.); 2 School of Food Science and Technology, Jiangnan University, Wuxi 214122, China; 3 Yangzhou Institute of Food Biotechnology, Jiangnan University, Yangzhou 225004, China; 4 National Engineering Research Center for Functional Food, Jiangnan University, Wuxi 214122, China; 5 Wuxi Translational Medicine Research Center and Jiangsu Translational Medicine Research Institute Wuxi Branch, Wuxi 214122, China

**Keywords:** probiotic, gut–brain axis, depression, microbiota, purine metabolism, xanthine oxidase

## Abstract

Depression is a common mood disorder that affects around 350 million people worldwide. We studied the effect of supplementation with *Lactobacillus* strains for the treatment of depression. Except for control group (*n* = 8), C57BL/6J mice were treated with *Lactobacillus* during six weeks of chronic unpredictable stress (depression group: *n* = 9, *Lactobacillus* intervention group: *n* = 7). *L. paracasei* CCFM1229 and *L. rhamnosus* CCFM1228 significantly reduced depressive behaviour in the forced swimming test and tail suspension test, significantly reduced anxiety behaviour in the open field test, and reduced anxiety behaviour in the marble burying test and light/dark box test. *L. paracasei* CCFM1229 and *L. rhamnosus* CCFM1228 significantly increased the brain serotonin and brain-derived neurotrophic factor concentrations, and CCFM1229 significantly decreased the serum corticosterone concentration, all of which are closely associated with the relief of depressive symptoms. Furthermore, CCFM1229 and CCFM1228 were shown to regulate purine metabolism in mice, as indicated by decreases in brain xanthine oxidase activity and an increase in liver adenosine deaminase activity. Anxiety- and depression-related indicators were significantly associated with xanthine oxidase activity in the cerebral cortex. The strains CCFM1229 and CCFM1228 reduced anxiety- and depression-related behaviour in a mouse model of chronic stress-induced depression, which may be achieved by regulating the activity of brain xanthine oxidase.

## 1. Introduction

Major depressive disorder (MDD) is a common mood disorder characterised by persistent depression, pessimism, frequent insomnia, mental distress, and, in severe cases, physical symptoms. Approximately 350 million people worldwide suffer from depression, and depression is a major cause of the global disease burden [1]. Currently, medications used to treat depression have inconsistent effects in different situations, and they are sometimes accompanied by unavoidable side effects such as nausea, vomiting, and diarrhoea [1]. A human adult’s intestinal tract contains 10^13^–10^14^ microbes, at least 10 times the number of human cells in the body. Altogether, intestinal microbes contain approximately 3 million genes, which is 150 times the number found in the human genome [2]. Diet, infection, disease status, and other factors can affect the composition of intestinal microbes, and abnormal microbiota can change behaviour, immunity, and endocrine function [2]. The intestinal tract contains more than 100 million neurons and serves as a gathering place for nerve, microbial, and immune cells [3]. The intestinal flora regulates both central nervous system and peripheral nerve function through effects on core nervous system processes, such as neurogenesis, synaptic plasticity, neurotransmitter signalling, neurodevelopment, and neuroinflammation, as well as microbial abnormalities, which are associated with autism spectrum disorder, depression, and Parkinson’s disease [4]. Bacteria are required for normal brain development and adult brain function [5,6]. Evidence from previous animal and clinical studies supports the crucial role of the gut microbiota in mood regulation, and depressive phenotypes can be inherited when the flora is transplanted from patients with major depression into normal mice [7].

Dietary regulation of the intestinal microbiota is a novel way to alleviate emotional disorders. Some probiotics show good anti-anxiety and antidepressant effects in areas such as neurotransmission, inflammation, hypothalamic–pituitary–adrenal (HPA) axis function, and the intestinal microbiota [7]. Previous studies have shown that some *Lactobacillus* strains can alleviate depression. *L. paracasei* PS23 can reverse anxiety- and depression-related behaviour caused by chronic corticosterone exposure [8]. *L. fermentum* PS150 was shown to reverse a decrease in serotonin levels in the brains of rats exposed to chronic mild stress, reduce the expression of *Ifng* and the plasma concentration of corticosterone, and restore the rats’ normal behaviour [9]. In rats, *L. helveticus* NS8 improved the behavioural and cognitive impairment induced by chronic stress, decreased the plasma concentrations of corticosterone (CORT) and adrenocorticotropic hormone (ACTH), restored the hippocampal levels of 5-hydroxytryptamine (5-HT) and norepinephrine (NE), and increased hippocampal expression of the gene encoding brain-derived neurotrophic factor (BDNF) [10]. *L. rhamnosus* JB-1 reduced stress-induced corticosterone levels and anxiety- and depression-related behaviours [11].

In addition, studies have shown that purine metabolism is disordered in patients with depression, and the activity of xanthine oxidase in the thalamus and putamen is significantly increased. It has been reported that xanthine oxidase activity can affect the nervous system. We speculate that brain xanthine oxidase activity may have a correlation with depression. However, there is no experimental animal evidence for a correlation between xanthine oxidase activity and depressive symptoms, and no reports of medicines or probiotics targeting xanthine oxidase to alleviate depression. Therefore, this study attempted to demonstrate the correlation between brain xanthine oxidase activity and depression, and also hoped that *Lactobacillus* that can alleviate depression by targeting xanthine oxidase can be obtained. Animal models currently mainly used in the study of depression include: chronic unpredictable stress model, learned helplessness model and chronic social defeat stress, behavioural despair model, maternal separation model, pharmacological depression model, and brain injury model. Among them, the chronic unpredictable stress model is widely used, which can simulate the generation of depressive symptoms in a natural state. The open field test, marble burying test, and light/dark box test are the classic behavioural tests used to measure anxiety in mice, and the tail suspension test and the forced swimming test are classic behavioural tests used to measure depression in mice. In this study, a mouse model of chronic unpredictable stress (CUS) was used to evaluate the effects of 13 *Lactobacillus* strains. It is hoped that this study can explain some of the possible ways that *Lactobacillus* can alleviate depression and provide effective dietary intervention strategies for the prevention and treatment of depression.

## 2. Materials and Methods

### 2.1. Probiotic Strains and Culture Conditions

The strains used in this study (*L. paracasei* 126L6, CCFM1229, 29L1, 4L3, *L. helveticus* 132M1, 8G3, Q7M66, 10M6, *L. rhamnosus* CCFM1131, CCFM1130, CCFM1228, *L. reuteri* CCFM1132, 11M59) were isolated from healthy human faeces or fermented food and stored in the food microbial culture library of Jiangnan University (Wuxi, China). Specific species and sources are shown in Supplementary Material Table S1. The study did not involve human experiments. Collecting faecal samples does not pose a foreseeable risk or discomfort to participants. Use of their stool samples was for public health purposes with written informed consent and the consent of the participants or their legal guardians. The strains were cultured in MRS medium under anaerobic conditions at 37 °C.

### 2.2. Animals and Treatment

Male adult C57BL/6J mice (6 weeks) were placed under controlled temperature (22 ± 1 °C) and humidity (55 ± 10%) for 12/12 h of light and dark cycle and allowed to obtain food and water freely. All procedures involving animals were carried out in strict accordance with the guidelines of the Institutional Animal Care and Use Committee (Wuxi, China). The scheme was approved by the ethics committee of experimental animals of Jiangnan University (Qualification number: JN.No 20210330c1080610(063)).

**Figure 1:**
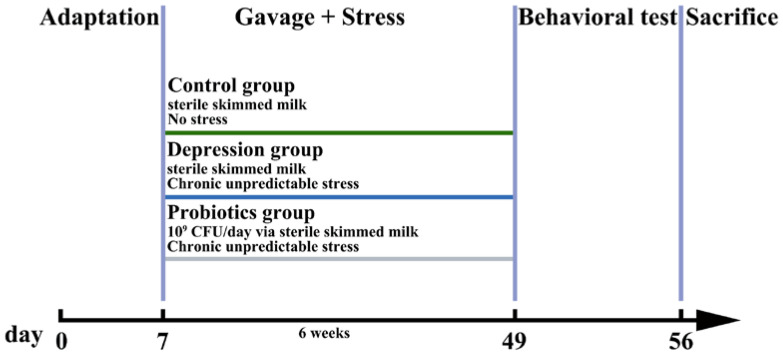
Animal experiment schedule. The whole experimental period was eight weeks, including one week of adaptation and six weeks of chronic stress. After the adaptation period, mice were gavaged until the day before mice were sacrificed. Behavioural tests were performed during the eighth week for all the mice.

Mice were randomly divided into different experimental groups according to body weight (control group (*n* = 8), depression group (*n* = 9), and *Lactobacillus* intervention group (*n* = 7)). Except for the control group, all mice received CUS intervention. CUS included random stress twice a day (see the detailed stress protocol in supplementary Materials) for six weeks. The control group and depression group were gavaged with sterile skimmed milk. The freeze-dried bacterial powder was suspended in sterile skimmed milk to prepare oral administration of *Lactobacillus* for mice. The concentration of surviving bacteria was 5 × 10^9^ CFU/mL. The gavage volume of each mouse is 200 μL. After the adaptation period, mice were gavaged until the day before mice were sacrificed. The animal experiment schedule is shown in Figure 1.

### 2.3. Behavioural Tests

Mice were moved to the testing room at least 30 min before all behavioural experiments. During the interval between events, the test equipment was regularly cleaned with 75% ethanol to avoid olfactory cues. Behavioural tests included open field test (OFT), marble burying test (MBT), light/dark box test (LDBT), tail suspension test (TST), and forced swimming test (FST) (see detailed scheme in Supplementary Materials).

### 2.4. Determination of Neurobiological Factors

The contents of 5-hydroxytryptamine (5-HT) were analysed by Waters 2695 Alliance HPLC with fluorescence detector (Waters 2475) and T3 Column (Waters XSelect HSS T3 Column, 100 Å, 5 µm, 4.6 mm × 250 mm, 1/pk). The determination method is provided by Min Guo and Chuanqi Chu. The mobile phase consisted of acetonitrile and PBS (pH 4.0) with an initial ratio of 5:95 (vol/vol) and gradient elution. The chromatographic column was heated to 30 °C and fluorescence detection was performed at 320 nm emission wavelength and 280 nm excitation wavelength. Serum corticosterone concentrations and brain-derived neurotrophic factor (BDNF) in the hippocampus were measured using an ELISA kit (Nanjing Sbjbio Biological Technology Co., Ltd., Nanjing, China).

### 2.5. Determination of Neuroregulatory Factors

The total RNA of the hippocampus and prefrontal lobe was extracted by FastPure Cell/Tissue Total RNA Isolation Kit (Vazyme Biotech Co., Ltd., Nanjing, China). cDNA was synthesised using a reverse transcription kit (Vazyme Biotech Co., Ltd., Nanjing, China). The concentration and purity of the synthesised cDNA samples were detected by NanoDrop 2000C (A260/A280 should be greater than 1.8) and stored at −80 °C for further use. Most primer sequences were obtained from Primerbank (Table 1). qRT-PCR was performed using the iTaq Univeral SYBR Green Supermix (BIO-RAD, Hercules, California, USA) on a BioRad-CFX384 fluorescent quantitative gene amplification instrument. Three parallel wells were set for each sample. Ct values were normalised to *Gapdh* and relative gene expression was calculated using the 2^−ΔΔCt^ method.

### 2.6. Gut Microbiota Analysis

As mentioned above, intestinal microbiota was analysed [12]. Total DNA was extracted from the colon contents near the cecum using the FastDNA^®^ Spin Kit for Stool (MP Biomedicals, Santa Ana, CA, USA) according to the manufacturer’s laboratory instructions. Universal primers (341F and 806R) were used to amplify the V3-V4 region of the 16S rRNA gene. PCR products were cut from 2.0% agarose gel, Qubit dsDNA HS Assay Kit (Life Technologies Corporation, Carlsbad, CA, USA) quantification. Libraries were constructed by a TruSeq DNA LT Sample Preparation Kit (Illumina, Santiago, CA, USA). Sequencing was performed using a MiSeq Reagent Kit (Illumina, Santiago, CA, USA) on the Illumina MiSeq PE300 Platform.

### 2.7. Determination of Purine Metabolism Indexes

Xanthine oxidase activity in the cerebral cortex and adenosine deaminase activity in the liver were measured using testing kits (Nanjing Jiancheng Bioengineering Institute, Nanjing, China). The level of Nrf2 in cerebral cortex was detected by ELISA kit (Jiangsu Meimian industrial Co., Ltd., Yancheng, China). The protein concentration of the cerebral cortex and liver was measured by the enhanced BCA protein assay kit (Beyotime Biotechnology, Shanghai, China).

### 2.8. Statistical Analysis

Statistical analysis was performed using SPSS 20 statistical software and Graphpad Prism 8.0.2. The data were evaluated for normal distribution and plotted with the mean of 95% Confidence Intervals (CI). The comparison between the control group and the model group used *t*-test. Significant differences were assessed using one-way ANOVA and Post Hoc Fisher’s Least Significant Difference (LSD) tests. The correlation analysis of microbiota was performed by QIIME2, and the function prediction of microbiota was performed by PICRUSt2. Linear Discriminant Analysis Effect Size (LEfSe) was performed online (http://huttenhower.sph.harvard.edu/galaxy/, accessed on 11 November 2021). The PCoA map of intestinal flora β diversity was created using ImageGP (http://www.ehbio.com/Cloud_Platform/front/#/, accessed on 8 November 2021). The diagram of intestinal flora composition at phylum level was created using OmicStudio Tools. OmicStudio tools were also used to mark the correlation between microbiota changes, KEGG function prediction, and depression phenotype on the cluster heat map (https://www.omicstudio.cn/tool, accessed on 27 December 2021).

## 3. Results

### 3.1. Some Lactobacillus Strains Can Alleviate Depressive Behaviour in Mice

**Figure 2:**
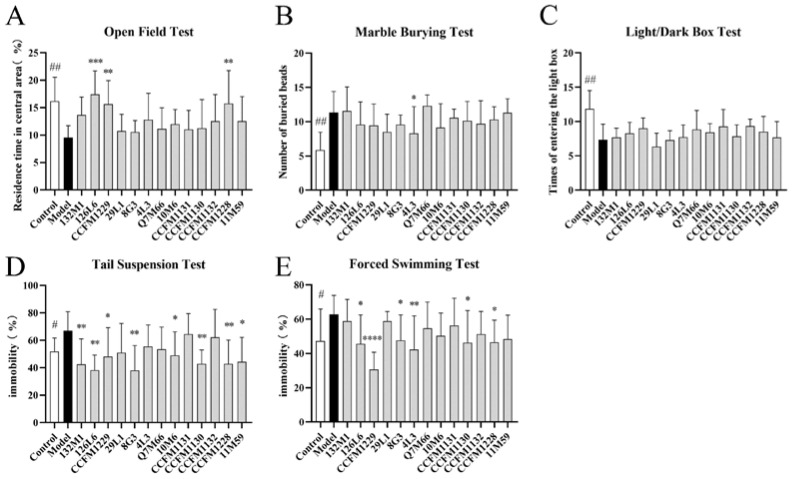
Behavioural tests. # *p* < 0.05; ## *p* < 0.01; * *p* < 0.05; ** *p* < 0.01; *** *p* < 0.001; **** *p* < 0.0001 comparing with depression group; *n* = 7–9.

The open field test, marble burying test, and light/dark box test were used to evaluate anxiety behaviour. In the open field test, mice treated with strains 126L6, CCFM1229, and CCFM1228 had a significantly increased residence time in the central area compared with the depression model group (Figure 2A). In the marble burying test, treatment with 4L3 significantly reduced the number of buried beads compared with the model group (Figure 2B). In the light/dark box test, mice treated with CCFM1131 entered the open chamber more frequently than mice in the depression model group (Figure 2C).

The tail suspension test and forced swimming test were used to evaluate depressive behaviour. In the tail suspension test, mice treated with strains 132M1, 126L6, CCFM1229, 8G3, 10M6, CCFM1130, CCFM1228, and 11M59 had significantly reduced immobility compared with mice in the depression model group (Figure 2D). In the forced swimming test, mice treated with 126L6, CCFM1229, 8G3, 4L3, CCFM1130, and CCFM1228 had significantly reduced immobility compared with mice in the depressive model group; of the listed strains, CCFM1229 was most effective (Figure 2E).

Strains 8G3, 4L3, and CCFM1130 yielded good effects in the two behavioural tests. Strains 126L6, CCFM1229, and CCFM1228 led to significant improvements in the three behaviours of depression and anxiety. Therefore, among the 13 strains tested, 126L6, CCFM1229 and CCFM1228 had the best anti-depressive effect. As mice treated with CCFM1229 significantly outperformed those in other treatment groups in the forced swimming test, we conclude that CCFM1229 has the best antidepressant effect.

### 3.2. The Effects of Lactobacillus on Neurobiology

**Figure 3:**
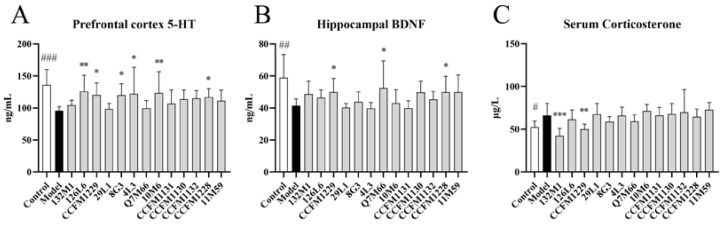
Effect of *Lactobacillus* on brain and neuroendocrine system. (**A**) Prefrontal cortex 5-HT levels. (**B**) BDNF concentration of the hippocampus. (**C**) Serum corticosterone levels. # *p* < 0.05; ## *p* < 0.01; ### *p* < 0.001; * *p* < 0.05; ** *p* < 0.01; *** *p* < 0.001 comparing with depression group; *n* = 7–9.

Treatment with strains 126L6, CCFM1229, 8G3, 4L3, 10M6, and CCFM1228 led to increased 5-HT levels in the prefrontal cortex (Figure 3A), and treatment with CCFM1229, Q7M66, and CCFM1228 led to increased BDNF levels in the hippocampus (Figure 3B). Treatment with 132M1 and CCFM1229 reduced corticosterone release induced by chronic stress (Figure 3C).

### 3.3. Unique Neuroregulatory Effects of Lactobacillus paracasei CCFM1229 and Lactobacillus rhamnosus CCFM1228

Five *Lactobacillus* strains with varying behaviours and biochemical indexes were selected to measure the related indexes of neuroregulatory factors. The five selected strains showed varying effects on mouse behaviour. Among the experimental *L. paracasei* strains, CCFM1229 and 4L3 could relieve anxiety and depression. CCFM1229 had the best performance, 4L3 had moderate performance, and 29L1 had the worst performance. Among the experimental *L. rhamnosus* strains, CCFM1228 yielded the best behavioural and biochemical results, whereas CCFM1131 yielded the worst results. *L. helveticus* and *L. reuteri* were not chosen because neither yielded optimal effects.

**Figure 4:**
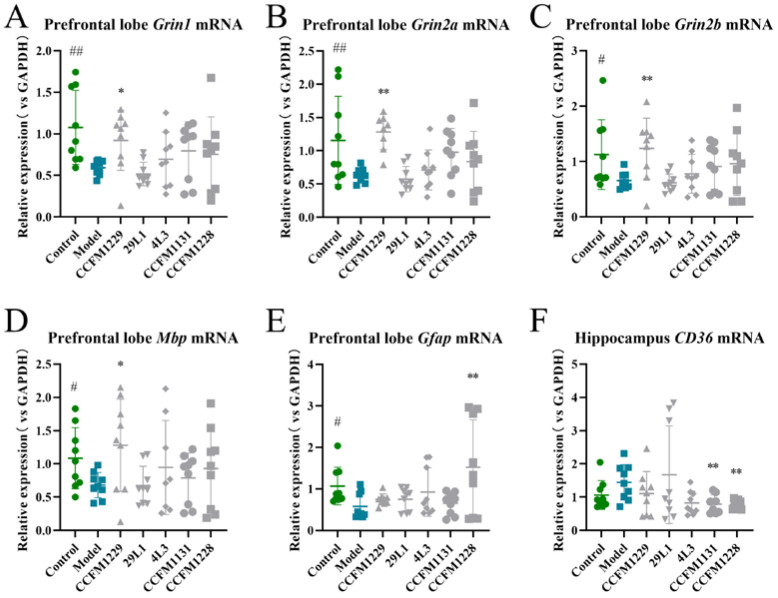
The expression of specific genes. # *p* < 0.05; ## *p* < 0.01; * *p* < 0.05; ** *p* < 0.01; comparing with depression group.

As shown in Figure 4A–C, *L. paracasei* CCFM1229 led to a significant upregulation of *Grin1*, *Grin2a,* and *Grin2b* mRNA expression in the depressed mice. The proteins encoded by these genes are important for synaptic plasticity. *L. paracasei* CCFM1229 led to a significant upregulation of *Mbp* mRNA expression in the depressed mice, suggesting that the structural and functional stability of myelin was maintained in the central nervous system (CNS) (Figure 4D).

*L. rhamnosus* CCFM1228 led to a significant upregulation of *Gfap* mRNA expression and enhanced astrocyte function in the depressed mice (Figure 4E). CD36 deficiency may affect depressive-like behaviour by changing the intestinal microbiota and inflammasome pathways. Hippocampal *CD36* mRNA expression was upregulated in the depressed mice, consistent with literature reports [13], whereas CCFM1228 significantly downregulated *CD36* mRNA expression (Figure 4F).

Regulation of the expression of these gene reflects the unique neuroregulatory effects of CCFM1229 and CCFM1228, which may be partly responsible for the behavioural effects observed with these strains.

### 3.4. Effects of Lactobacillus on Microbiome Disorders Caused by Chronic Stress

**Figure 5:**
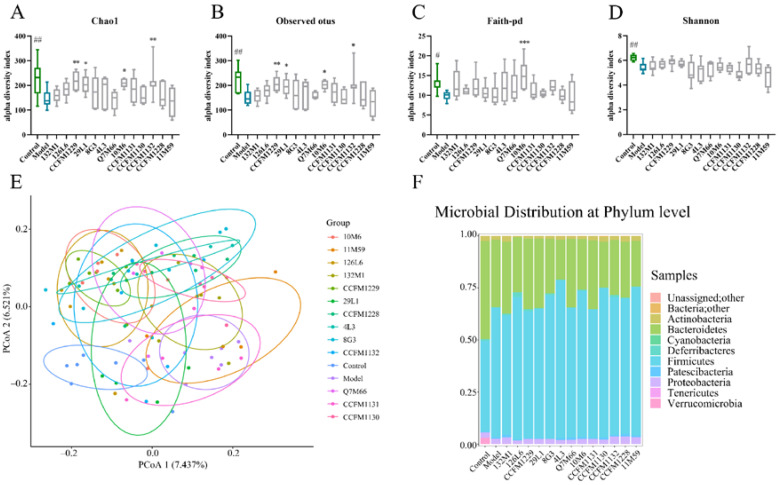
Gut microbiota analysis. (**A**–**D**) Alpha diversity indicated by Chao 1 index, observed OTUS, Faith-PD and Shannon index. (**E**) PCoA. (**F**) Microbial distribution at the phylum level. # *p* < 0.05; ## *p* < 0.01; * *p* < 0.05; ** *p* < 0.01; *** *p* < 0.001 comparing with depression group; *n* = 7–9.

Next, the effects of *Lactobacillus* strains on intestinal microecology were studied by 16S rRNA amplicon sequencing. Compared with the control group, significant changes in the intestinal microbial structure were observed in chronically stressed mice. Alpha diversity was evaluated using the Chao1 index, observed operational taxonomic units (OTUs), Faith-PD index, and Shannon index. Following specific probiotic treatment, the Chao1 index and observed OTUs increased significantly and the Faith-Pd index increased, whereas the Shannon index did not change significantly (Figure 5A–D). Using principal co-ordinates analysis to reflect beta diversity, the control and model groups were significantly separated, and a new homeostasis was established after some strain interventions (Figure 5E).

At the phylum level, the abundance of Bacteroidetes and Verrucomicrobia was significantly decreased in the depressed mice, whereas the abundance of Firmicutes was significantly increased (Figure 5F). All of these results indicate significant structural changes in the intestinal microbiota from before to after the probiotic intervention.

**Figure 6:**
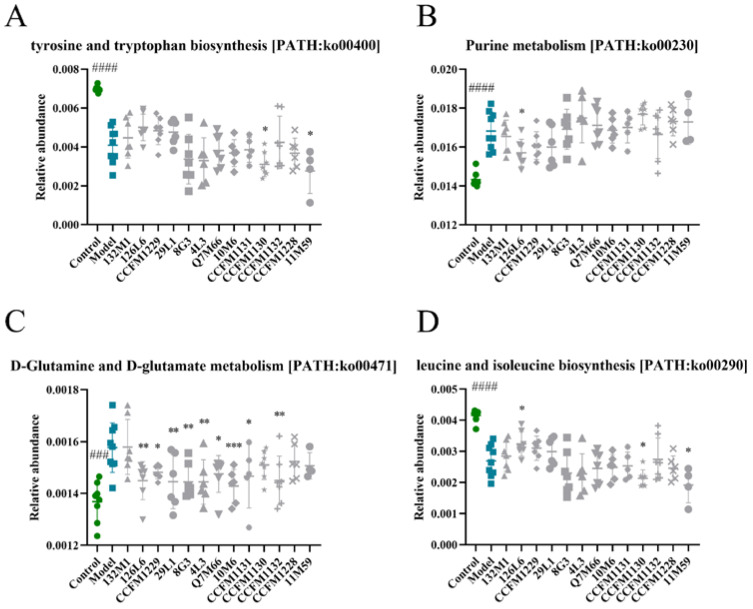
KEGG function prediction of 16S rRNA by PICRUSt2. (**A**) Tyrosine and tryptophan biosynthesis. (**B**) Purine metabolism. (**C**) D-glutamine and D-glutamate metabolism. (**D**) Leucine and isoleucine biosynthesis. ### *p* < 0.001; #### *p* < 0.0001; * *p* < 0.05; ** *p* < 0.01; *** *p* < 0.001; comparing with depression group.

PICRUSt2 was used to predict the function of the microbiota. According to the Kyoto Encyclopedia of Genes and Genomes (KEGG), LEfSe analysis revealed differences in 139 functional categories between the control and depressed mice (Supplementary material Figure S1), some of which may be related to the mode and intensity of modelling. We focused on tyrosine and tryptophan biosynthesis, purine metabolism, D-glutamine and D-glutamate metabolism, and leucine and isoleucine biosynthesis. These functions were altered significantly in the depressed mice, and specific strain treatments were able to reverse these changes (Figure 6). In the Supplementary Material, Figure S2 depicts the changes in aminoacyl-tRNA biosynthesis, cell growth, DNA repair and recombinant proteins, base excision repair, and nucleotide excision repair. As shown in Figure 6A, the strains 126L6, CCFM1229, and 29L1 led to increases in tyrosine and tryptophan biosynthesis, but the effect was not statistically significant. The strains CCFM1130 and 11M59 led to a significant reduction in tyrosine and tryptophan biosynthesis. As shown in Figure 6B, 126L6 significantly regulated purine metabolism. As shown in Figure 6C, multiple strains with different antidepressant effects significantly regulated D-glutamine and D-glutamate metabolism. As shown in Figure 6D, significant increases in leucine and isoleucine biosynthesis were observed in the 126L6 intervention group, whereas significant decreases were observed in the CCFM1130 and 11M59 groups.

### 3.5. Effect of Lactobacillus on Purine Metabolism

**Figure 7:**
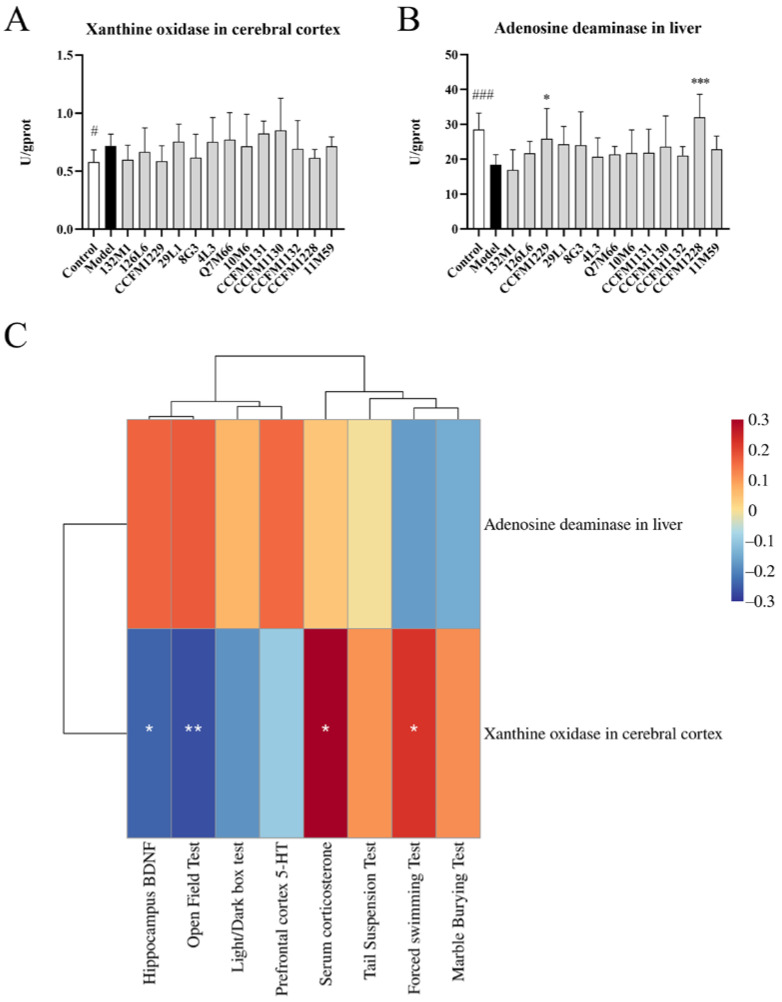
Effect of *Lactobacillus* on Purine metabolism. (**A**) Xanthine oxidase activity in the cerebral cortex. (**B**) Adenosine deaminase activity in liver. (**C**) Correlation Heatmap of depression related indexes and purine metabolism indexes. # *p* < 0.05; ### *p* < 0.001; * *p* < 0.05; ** *p* < 0.01; *** *p* < 0.001.

Next, the effects of *Lactobacillus* supplementation on purine metabolism in various organs were determined. Treatment with 132M1, CCFM1229, 8G3 and CCFM1228 decreased xanthine oxidase activity in the cerebral cortex (Figure 7A). Compared with the model group, CCFM1229 and CCFM1228 significantly increased adenosine deaminase activity in the liver (Figure 7B). Except in the control group, there were no significant differences in the levels of Nrf2 among the model group and other *Lactobacillus* treated groups (Figure S3).

Analysis of correlations between the purine metabolism indices and depression indices showed that xanthine oxidase activity in the cerebral cortex was significantly correlated with the central area residence time in the open field test, immobility time in the forced swimming test, the serum corticosterone concentration and hippocampal BDNF level (Figure 7C). In summary, disordered purine metabolism caused by chronic unpredictable stress is linked to depressive and anxiety symptoms.

### 3.6. Depression Indices Were Closely Related to Changes in the Microflora and KEGG Functionality

**Figure 8:**
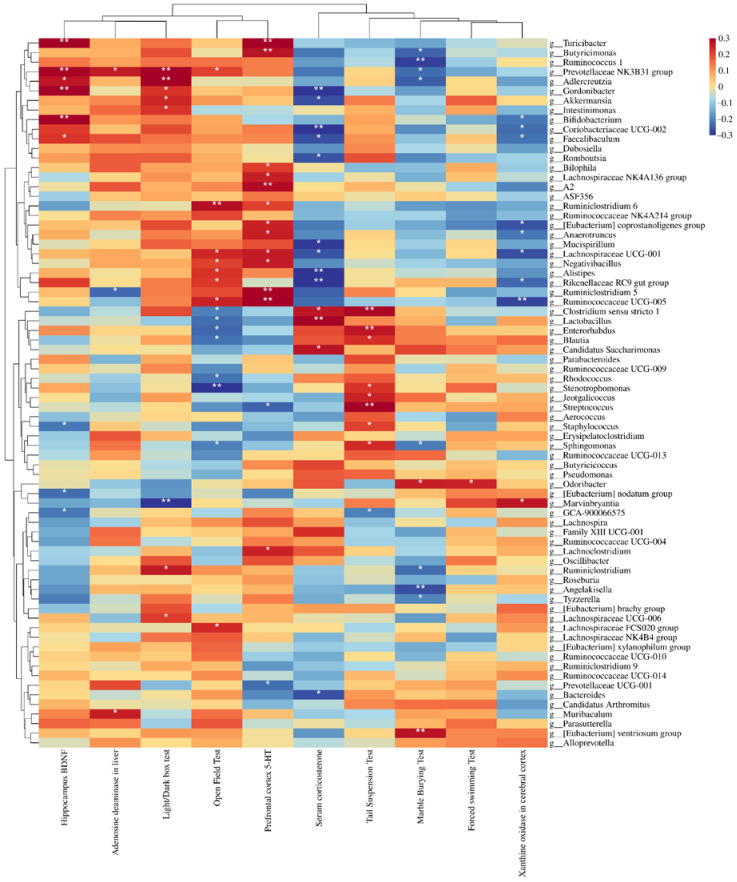
Correlation between bacterial abundance and behavioural indexes, neurobiological indexes, and purine metabolism indexes. * *p* < 0.05; ** *p* < 0.01.

**Figure 9:**
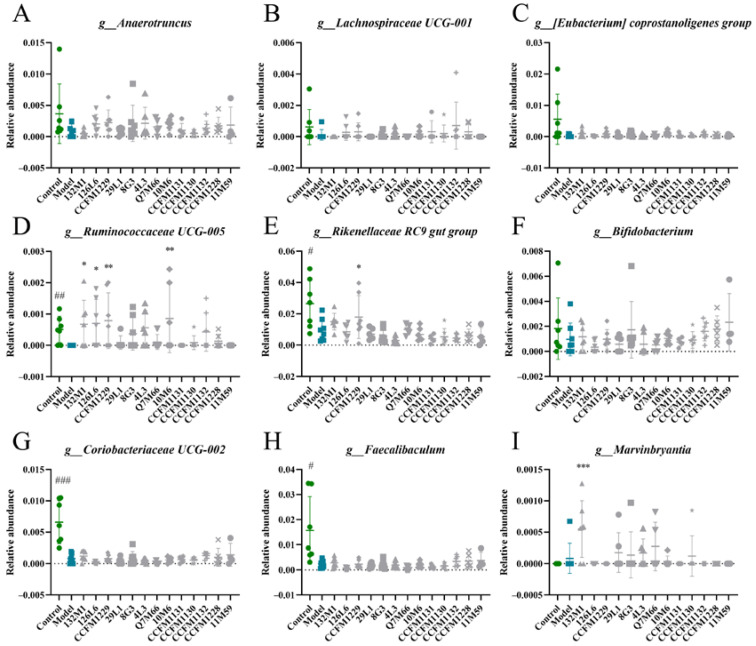
Relative abundance of bacteria associated with xanthine oxidase activity in cerebral cortex. (**A**) *Anaerotruncus*. (**B**) *Lachnospiraceae UCG-001*. (**C**) *[Eubacterium] Coprostanoligenes group.* (**D**) *Ruminococcaceae UCG-005.* (**E**) *Rikenellaceae RC9 gut group.* (**F**) *Bifidobacterium*. (**G**) *Coriobacteriaceae UCG-002.* (**H**) *Faecalibaculum*. (**I**) *Marvinbryantia*. # *p* < 0.05; ## *p* < 0.01; ### *p* < 0.001; * *p* < 0.05; ** *p* < 0.01; *** *p* < 0.001 comparing with depression group.

Figure 8 shows the correlations between bacterial abundance and behavioural, neurobiological, and purine metabolism indices. All phenotypic indicators of depression were significantly correlated with the abundance of intestinal bacteria. We noted that at the genus level, the abundance of *Anaerotruncus*, *Lachnospiraceae UCG-001*, *[Eubacterium] Coprostanoligenes Group*, *Ruminococcaceae UCG-005*, *Rikenellaceae RC9 gut group*, *Bifidobacterium*, *Coriobacteriaceae UCG-002*, and *Faecalibaculum* was significantly negatively correlated with xanthine oxidase activity in the cerebral cortex. The abundance of *Marvinbryantia* was positively correlated with xanthine oxidase activity in the cerebral cortex. Interestingly, all of these genera were associated with depression-related indices, suggesting a close relationship between xanthine oxidase activity in the cerebral cortex and the depression phenotype, mediated by the intestinal flora. Figure 9 shows the relative abundance of these bacteria in each group.

As shown in Figure 9, the abundance of *Marvinbryantia* was upregulated in mice with depression, contrary to the trends observed for other bacterial genera. The relative abundance of *Ruminococcaceae UCG-005*, *Rikenellaceae RC9 gut group*, *Coriobacteriaceae UCG-002* and *Faecalibaculum* differed between the control group and the model group. Some *Lactobacillus* species had significant regulatory effects on the relative abundance of *Ruminococcaceae UCG-005*, *Rikenellaceae RC9 gut group*, and *Marvinbryantia*. However, the experimental *Lactobacillus* strains did not significantly regulate the abundance of *Anaerotruncus*, *Lachnospiraceae UCG-001*, *[Eubacterium] Coprostanoligenes*, *Bifidobacterium*, *Coriobacteriaceae UCG-002*, and *Faecalibaculum*.

**Figure 10:**
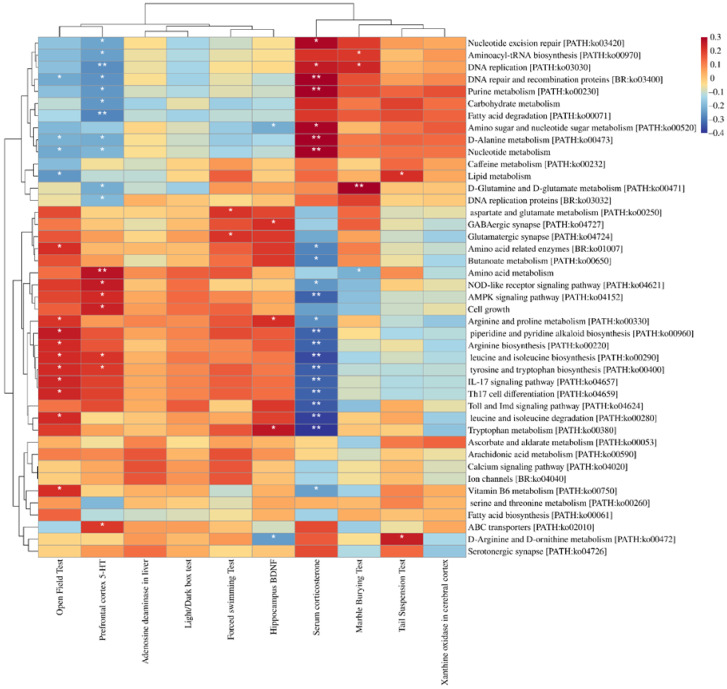
Correlation between KEGG annotated microbial function and depression indicators. * *p* < 0.05; ** *p* < 0.01.

Figure 10 shows that all depression indices were significantly correlated with KEGG-annotated microbial functionality. Purine metabolism was significantly positively correlated with the serum concentration of corticosterone and negatively correlated with the 5-HT level in the prefrontal cortex.

## 4. Discussion

Probiotics elicit mood changes via the gut–brain–microbial axis. We screened 13 strains for their potential antidepressant effects in a mouse model of depression induced by chronic unpredictable stress. Anxiety-related behaviour was evaluated using the open-field, marble-burying, and light-and-dark box tests; depressive behaviour was evaluated using the forced swimming and tail suspension tests. We further used neurobiological indicators to assist in the evaluation of these strains’ potential antidepressant effects. The results showed that 126L6, CCFM1229, and CCFM1228 effectively relieved depression and anxiety symptoms in mice.

The monoamine hypothesis states that depression is caused by a decline in brain monoamine function, and is based on the efficacy of single amine drugs as antidepressants, selective serotonin reuptake inhibitors (SSRIs), which are mainstream antidepressant drugs, and antidepressants that induce a rapid increase in the level of synaptic monoamines and produce secondary neural plasticity changes [14]. BDNF is an important plasticity-related protein that promotes neuronal growth, development, and survival [15] and is crucial for axonal growth, neuronal survival, and synaptic plasticity. The HPA axis is an important part of the neuroendocrine system, and disorders of this axis are related to mood disorders [12]. In severe cases, glucocorticoids can damage neurogenesis in the hippocampus, destroy synaptic plasticity, and even cause neurotoxicity [16]. The *Lactobacillus* strains 126L6, CCFM1229, and CCFM1228 increased 5-HT levels in the prefrontal cortex and BDNF levels in the hippocampi of mice in this study, and CCFM1229 reduced serum corticosterone concentrations, suggesting that treatment with 126L6, CCFM1229, and CCFM1228 can improve the core biochemical depression indices. The N-methyl-D-aspartate receptors (NMDARs) are a class of ionic glutamate receptors. The number and subunit composition of synaptic NMDARs do not remain constant but change in a cell- and synapse-specific manner during development in response to neuronal activity and sensory experience [17]. Maladjustment of the bidirectional regulation of NMDARs may lead to neuropsychiatric diseases [17]. Our study suggests that CCFM1229 increases expression of the gene encoding NMDAR and may regulate glutamate metabolism by increasing NMDAR function, which is linked to depression. The disruption of myelin, oligodendrocytes, oligodendrocyte precursor cells, neural support, and neuroregulation may form the basis of MDD and stress-related disorders. Myelin loss and oligodendrocyte dysfunction may be related to the pathogenesis of depression, a supposition that is supported by the co-occurrence of demyelination disorder and depression in humans and rodents [18]. Treatment with strain CCFM1229 helps stabilise the nervous system by upregulating *Mbp* mRNA expression. Chronic stress promotes astrocyte malnutrition in the prefrontal cortex, which is related to behavioural despair [19]. The strain CCFM1228 was shown to regulate *Gfap* mRNA expression, which may promote astrocyte proliferation or support astrocyte function. These neuromodulatory effects may explain the antidepressant effects of CCFM1229 and CCFM1228. Depression is linked to tyrosine and tryptophan biosynthesis and D-glutamine and D-glutamate metabolism. The probiotics tested in this study were shown to regulate tyrosine and tryptophan biosynthesis, although these effects were not significant. Some strains significantly regulated D-glutamine and D-glutamate metabolism; however, some of these strains had poor antidepressant activity, indicating that D-glutamine and D-glutamate metabolism is not the only function associated with the depression phenotype. Leucine and isoleucine biosynthesis was also significantly reduced in the microflora of depressed mice. Because leucine is easily converted to glucose, it helps to regulate blood glucose concentrations. Leucine deficiency can cause symptoms similar to hypoglycaemia, such as headache, dizziness, fatigue, depression, and confusion. Intervention with some probiotics can significantly upregulate leucine and isoleucine biosynthesis, which may explain why these strains improve mood.

The predicted results of a microbiota functional analysis indicate significant changes in purine metabolism during depression. A purine cycle disorder may be associated with MDD; specifically, changes in purine metabolism may lead to an accumulation of circulating xanthines in MDD patients [20]. These patients have abnormal purine metabolism and exhibit differences in xanthine, hypoxanthine, adenosine, inosine, and uric acid concentrations compared with healthy controls. Purines and monoamines can be metabolised concurrently in the brain [21,22]. In CD4^+^ T cells with fragmented mitochondria, glucose is not metabolised via the normal glycolysis pathway; rather, large amounts of purines are synthesised via the pentose phosphate pathway and released from the cells, and xanthines produced in this manner can easily cross the blood–brain barrier and reach the amygdala [23]. In mice, xanthines act on oligodendrocytes in the amygdala through purine receptors on the cell surface, causing abnormal oligodendrocyte activation and proliferation, and ultimately, hyperactivation of local neurons in the fear centre, leading to severe anxiety behaviours [23]. Our study is the first to demonstrate that anxiety indices (the central area residence time in the open field test) and depression indices (the immobility time in the forced swimming test, serum corticosterone concentration, and hippocampal BDNF level) are significantly associated with xanthine oxidase activity in the cerebral cortex. Treatment with the strains CCFM1229 and CCFM1228 alleviated depression in mice by decreasing xanthine oxidase activity in the cerebral cortex. The KEGG analysis showed that 126L6 significantly downregulated purine metabolism but had a limited ability to regulate xanthine oxidase activity in the brain, suggesting that this strain may alleviate depression by regulating other aspects of purine metabolism. In MDD patients, xanthine oxidase activity was shown to increase significantly in the thalamus and putamen, suggesting that oxidative stress plays a key role in some brain regions of recurrent depression [24]. Xanthine and xanthine oxidase generate free radicals and superoxide anions, which produce oxidative stress and lead to cell damage and death [25]. Reactive oxygen species produced by the interaction of xanthine with xanthine oxidase increase the release of the neurotransmitter L-glutamate [26,27]. Glutamate is the most abundant neurotransmitter in the brain and a precursor to gamma-aminobutyric acid. Glutamate, which is highly excitable, can produce neurotoxicity; this process is observed in many mental disorders and can lead to brain damage in severe cases. Xanthine oxidase is associated with ROS production and Nrf2 regulation. Nrf2 has been shown to reduce depression. However, from the present result, the regulation of xanthine oxidase activity by lactic acid bacteria does not appear to affect the level of Nrf2. Some probiotics tested in this study regulate glutamate metabolism via the intestinal microflora, which may explain why probiotics alleviate depressive symptoms. CCFM1229 and CCFM1228 regulate purine metabolism by improving the intestinal microflora and may thus affect xanthine oxidase activity in the brain. The relative abundance of *Anaerotruncus*, *Lachnospiraceae UCG-001*, *[Eubacterium] Coprostanoligenes Group*, *Ruminococcaceae UCG-005*, *Rikenellaceae RC9 gut group*, *Bifidobacterium*, *Coriobacteriaceae UCG-002*, *Faecalibaculum*, and *Marvinbryantia* were significantly correlated with xanthine oxidase activity in the cerebral cortex. Sea buckthorn pulp oil was shown to have immunoenhancing effects in suppressed mice by cyclophosphamide-induced immunosuppression and increases the abundance of *Anaerotruncus* [28]. Glycine was shown to protect against lipopolysaccharide-induced intestinal injury and improve the abundance of *Anaerotruncus* [29]. *Lachnospiraceae UCG-001* is an acetic acid-producing bacterial species associated with health [30,31]. Inflammatory factors such as interleukin-12p40, interferon-gamma, and DR5 were negatively associated with the abundance of specific bacterial genera, namely *Ruminococcaceae UCG-009*, *Lachnospiraceae UCG-001*, and *Akkermansia* [32]. A specific dehydrogenase distinct from nicotinic acid hydroxylase was induced during the growth of *Eubacterium barkeri* on xanthine [33], implying that *Eubacterium* can regulate purine metabolism. *E. coprostanoligenes* can reduce cholesterol [34] and regulate lipid metabolism. The abundance of *[Eubacterium] Coprostanoligenes group* was reported to be negatively correlated with the severity of anxiety [35], which is consistent with our results. Compound probiotics improved cognitive performance in an acute stress environment and enhanced the abundance of *Ruminococcaceae UCG-005* [36]. A high-fat diet increased the abundance of the *Rikenellaceae RC9 gut group* [37], the members of which are sometimes classified as harmful bacteria due to increases in abundance in some metabolic diseases. However, some studies have reported a useful role for the *Rikenellaceae RC9 gut group*, the abundance of which was shown to decrease significantly in mice with type 2 diabetic nephropathy [38], H7N9-infected mice [39] and rats with hypertriglyceridemia-related acute necrotising pancreatitis [40]. The *Rikenellaceae RC9 gut group* has been negatively correlated with serum lipid, glucose, and insulin concentration [41] but positively correlated with the cecal acetic acid and isobutyric acid levels in mice with type 2 diabetic nephropathy [41]. Some specific strains of *Bifidobacterium* have been shown to alleviate depression [42,43], including the ‘commercialised’ *B. longum* R0175, a well-known antidepressant lactic acid bacteria. The abundance of *Faecalibaculum*, *Lactobacillus*, and *Coriobacteriaceae UCG-002* in the intestinal microflora was correlated negatively with alanine aminotransferase, aspartate aminotransferase, alkaline phosphatase, and malondialdehyde levels [44]. Chronic mild stress was shown to significantly increase the abundance of *Marvinbryantia* [45], which is consistent with our results.

This study has some limitations. Hence, further research is required to determine the specific target substances, modes of action, metabolism kinetics, and specific mechanisms by which probiotics reduce xanthine oxidase activity in the cerebral cortex.

## 5. Conclusions

The findings of this study suggest that intervention with *L. paracasei* CCFM1229 and *L. rhamnosus* CCFM1228 can alleviate in mice the behavioural and neurological changes induced by chronic stress. The strain CCFM1229 significantly upregulated the mRNA levels of *Grin1*, *Grin2a*, and *Grin2b* in the depressed mice, which is of great significance in terms of synaptic plasticity. CCFM1229 and CCFM1228 significantly upregulated *Mbp* mRNA expression in depressed mice, and maintained the stability of CNS myelin structure and function. The strain CCFM1228 upregulated *Gfap* mRNA expression and supported astrocyte function. We demonstrated for the first time that anxiety indices (the central area residence time in the open field test) and depression indices (the immobility time in the forced swimming test, serum corticosterone concentration, and hippocampal BDNF level) are significantly associated with xanthine oxidase activity in the cerebral cortex. Treatment with the strains CCFM1229 and CCFM1228 reduced anxiety- and depression-related behaviours in a chronic stress depression model, possibly by increasing the relative abundance of bacterial genera such as *Anaerotruncus*, *Lachnospiraceae UCG-001*, *Ruminococcaceae UCG-005*, *Rikenellaceae RC9 gut group*, and *Bifidobacterium* to regulate xanthine oxidase activity in the brain.

## Figures and Tables

**Table 1:** Primer sequence.

Gene	Sequence (5′ - > 3′)	PrimerBank ID
*Mbp*	F-5′-GGCGGTGACAGACTCCAAG-3′	51333a1
	R-5′-GAAGCTCGTCGGACTCTGAG-3′	
*Grin1*	F-5′-TCCCAACGACCACTTCACTC-3′	294997255c3
	R-5′-AGTAGATGGACATTCGGGTAGTC-3′	
*Grin2a*	F-5′-ACGTGACAGAACGCGAACTT-3′	41680704c1
	R-5′-TCAGTGCGGTTCATCAATAACG-3′	
*Grin2b*	F-5′-GCCATGAACGAGACTGACCC-3′	6680099a1
	R-5′-GCTTCCTGGTCCGTGTCATC-3′	
*Gfap*	F-5′-GGGGCAAAAGCACCAAAGAAG-3′	32480796a1
	R-5′-GGGACAACTTGTATTGTGAGCC-3′	
*CD36*	F-5′-ATGGGCTGTGATCGGAACTG-3′	227116348c1
	R-5′-GTCTTCCCAATAAGCATGTCTCC-3′	
*Gapdh*	F-5′-TCCTGCACCACCAACTGCT-3′	--
	R-5′-GTCAGATCCACGACGGACACA-3′	

## Data Availability

The datasets generated and analysed during the current study are available from the corresponding author on reasonable request.

## Supplementary Materials

The following supporting information can be downloaded at: https://www.mdpi.com/article/10.3390/nu14061294/s1, Table S1: Genus and sources of probiotics; Figure S1: LEfSe analysis revealed 139 different functional categories between control and depressed mice; Figure S2: KEGG function prediction of 16S rRNA by PICRUSt2.
