# Supplementary material for: Lactobacillus paracasei CCFM1229 and Lactobacillus rhamnosus CCFM1228 Alleviated Depression- and Anxiety-Related Symptoms of Chronic Stress-Induced Depression in Mice by Regulating Xanthine Oxidase Activity in the Brain
Source: Nutrients. 2022 Mar 18;14(6):1294. doi: 10.3390/nu14061294 (PMC8953819; doi:10.3390/nu14061294)
Supplement: Supplementary file 1 [file nutrients-14-01294-s001.zip › nutrients-1558924-supplementary.pdf]

Supplementary materials

Materials and methods

1. Modeling scheme of chronic unpredictable stress

Chronic unpredictable stress includes the following 10 stimuli :(1) fasting for 24 hours; (2) No water + empty bottle stimulation for 24h; (3) The tail was clamped for 3min, repeated three times; (4) Wet bedding for 24h; (5) Brake restraint for 6h; (6) Forced swimming for 15 minutes; (7) Solitary rearing for 24 hours; (8) Continuous illumination for 24h; (9) no bedding material; (10) Crowded stimulation. Avoid biorhythms and apply 2 of them randomly each day.

2. Detailed plan of the behavioral experiments

(1) Open field test

The experiment was carried out in a quiet environment. After the animals were put into the open field center, timing and video recording were immediately started, and the video recording was stopped after 10min observation. When changing animals, in order to avoid affecting the results of the next test, it is necessary to clean the inner wall and bottom surface of the open field first to remove the information left by the last animal (such as urine, feces, smell, etc.). EthoVision software was used for digital analysis of the video, and the percentage of time the mice stayed in the central area of the open field was calculated :(Residence time in the central area of the open field/total monitoring time) ×100%.

(2) Marble burying test

Lay a 5 cm thick corncob mat in the cage, and smooth it out. Put 20 glass beads (15 mm in diameter) into corn cob bedding, divided into five rows, each row of 4, regular arrangement. The mice were placed in the cage and videotaped for 30 min. Pave the pad and reuse it. Count the number of buried

glass beads (buried volume more than 50%).

(3) Light/dark box test

The mice were placed in the open box and placed in the center compartment of the shuttle box. The EthoVision real-time monitoring system was used to record the activity of the mice for 5min, and the number of mice entering the open box was calculated.

(4) Tail suspension test

The tail suspension test is similar to the forced swimming experiment in that it is a model of behavioral desperation. The rear 1/3 of the mouse tail was fixed with adhesive tape, suspended on the bracket, the head was 30cm away from the table, and the camera was taken. The background of the camera was in obvious contrast with the mouse hair color. The mice were timed for 5min and stopped. The immobility time of the mice was statistically analyzed by the small animal behavior analysis software. Among them, immobile state refers to the animal giving up the active struggle, in the state of complete immobility.

(5) Forced swimming test

About 2.2L of water was stored in the 3L experimental bucket at a temperature of 24±1℃. 24 hours before the formal experiment, each mouse underwent adaptive swimming training for 10 minutes. In the formal experiment, each mouse underwent a six-minute swimming test. EthoVision real-time monitoring system was used for video recording and analysis, and the Immobility time of mice was calculated (Immobility was defined as immobility of limbs or slight movement of hind limbs).

Supplementary results

Table S1 Genus and sources of probiotics.

Text number	Original number	Genus	Source
132M1	V-GZTR-132-M1	*L. helveticus*	pickles
126L6	VCQBB4126L6	*L. paracasei*	pickle water
CCFM1229	VCQYB7171M7	*L. paracasei*	pickle water
29L1	FZJHZD29L1	*L. paracasei*	adult feces
8G3	8G3	*L. helveticus*	wooden house, backer, outdoor
4L3	FHNFQ4L3	*L. paracasei*	baby feces
Q7M66	DQHXNQ07 M66	*L. helveticus*	Qula
10M6	FSDJN10M6	*L. helveticus*	adult feces
CCFM1131	FHuBei281	*L. rhamnosus*	feces of long-lived elderly
CCFM1130	FBJCY31	*L. rhamnosus*	baby feces
CCFM1132	FZJTZ20M3	*L. reuteri*	adult feces
CCFM1228	FAHWH30L7	*L. rhamnosus*	feces from healthy old people
11M59	DYNDL11M59	*L. reuteri*	sweet pulp

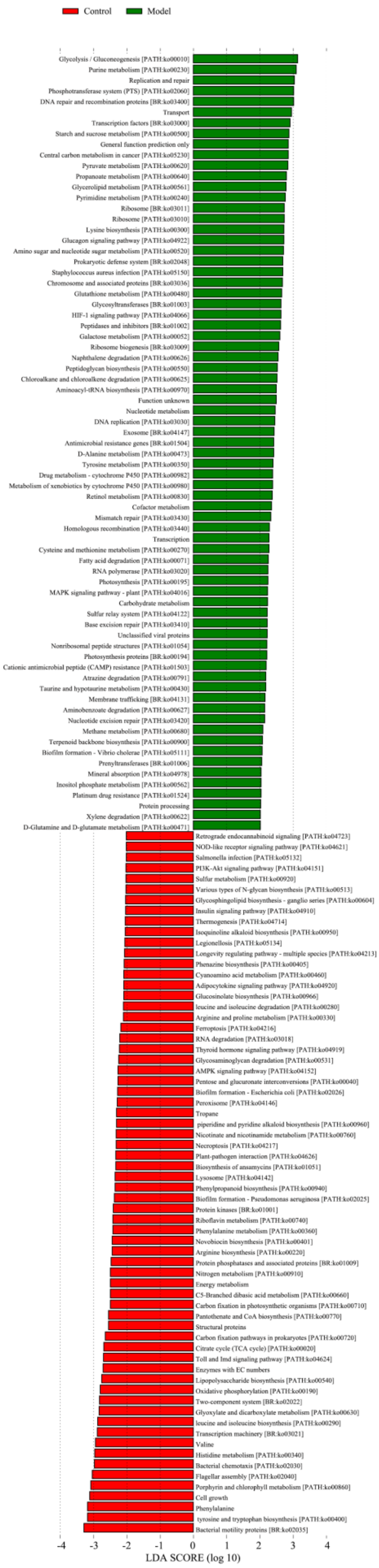
Control
Model
Glycolysis / Gluconeogenesis [PATH:ko00010]
Purine metabolism [PATH:ko00230]
Replication and repair
Phosphotransferase system (PTS) [PATH:ko02060]
DNA repair and recombination proteins [BR:ko03400]
Transport
Transcription factors [BR:ko03000]
Starch and sucrose metabolism [PATH:ko00500]
General function prediction only
Central carbon metabolism in cancer [PATH:ko05230]
Pyruvate metabolism [PATH:ko00620]
Propanoate metabolism [PATH:ko00640]
Glycerolipid metabolism [PATH:ko00561]
Pyrimidine metabolism [PATH:ko00240]
Ribosome [BR:ko03011]
Ribosome [PATH:ko03010]
Lysine biosynthesis [PATH:ko00300]
Glucagon signaling pathway [PATH:ko04922]
Amino sugar and nucleotide sugar metabolism [PATH:ko00520]
Prokaryotic defense system [BR:ko02048]
Staphylococcus aureus infection [PATH:ko05150]
Chromosome and associated proteins [BR:ko03036]
Glutathione metabolism [PATH:ko00480]
Glycosyltransferases [BR:ko01003]
HIF-1 signaling pathway [PATH:ko04066]
Peptidases and inhibitors [BR:ko01002]
Galactose metabolism [PATH:ko00052]
Ribosome biogenesis [BR:ko03009]
Naphthalene degradation [PATH:ko00626]
Peptidoglycan biosynthesis [PATH:ko00550]
Chloroalkane and chloroalkene degradation [PATH:ko00625]
Aminoacyl-tRNA biosynthesis [PATH:ko00970]
Function unknown
Nucleotide metabolism
DNA replication [PATH:ko03030]
Exosome [BR:ko04147]
Antimicrobial resistance genes [BR:ko01504]
D-Alanine metabolism [PATH:ko00473]
Tyrosine metabolism [PATH:ko00350]
Drug metabolism - cytochrome P450 [PATH:ko00982]
Metabolism of xenobiotics by cytochrome P450 [PATH:ko00980]
Retinol metabolism [PATH:ko00830]
Cofactor metabolism
Mismatch repair [PATH:ko03430]
Homologous recombination [PATH:ko03440]
Transcription
Cysteine and methionine metabolism [PATH:ko00270]
Fatty acid degradation [PATH:ko00071]
RNA polymerase [PATH:ko03020]
Photosynthesis [PATH:ko00195]
MAPK signaling pathway - plant [PATH:ko04016]
Carbohydrate metabolism
Sulfur relay system [PATH:ko04122]
Base excision repair [PATH:ko03410]
Unclassified viral proteins
Nonribosomal peptide structures [PATH:ko01054]
Photosynthesis proteins [BR:ko00194]
Cationic antimicrobial peptide (CAMP) resistance [PATH:ko01503]
Atrazine degradation [PATH:ko00791]
Taurine and hypotaurine metabolism [PATH:ko00430]
Membrane trafficking [BR:ko04131]
Aminobenzoate degradation [PATH:ko00627]
Nucleotide excision repair [PATH:ko03420]
Methane metabolism [PATH:ko00680]
Terpenoid backbone biosynthesis [PATH:ko00900]
Biofilm formation - Vibrio cholerae [PATH:ko05111]
Prenyltransferases [BR:ko01006]
Mineral absorption [PATH:ko04978]
Inositol phosphate metabolism [PATH:ko00562]
Platinum drug resistance [PATH:ko01524]
Protein processing
Xylene degradation [PATH:ko00622]
D-Glutamine and D-glutamate metabolism [PATH:ko00471]
Retrograde endocannabinoid signaling [PATH:ko04723]
NOD-like receptor signaling pathway [PATH:ko04621]
Salmonella infection [PATH:ko05132]
PI3K-Akt signaling pathway [PATH:ko04151]
Sulfur metabolism [PATH:ko00920]
Various types of N-glycan biosynthesis [PATH:ko00513]
Glycosphingolipid biosynthesis - ganglio series [PATH:ko00604]
Insulin signaling pathway [PATH:ko04910]
Thermogenesis [PATH:ko04714]
Isoquinoline alkaloid biosynthesis [PATH:ko00950]
Legionellosis [PATH:ko05134]
Longevity regulating pathway - multiple species [PATH:ko04213]
Phenazine biosynthesis [PATH:ko00405]
Cyanoamino acid metabolism [PATH:ko00460]
Adipocytokine signaling pathway [PATH:ko04920]
Glucosinolate biosynthesis [PATH:ko00966]
leucine and isoleucine degradation [PATH:ko00280]
Arginine and proline metabolism [PATH:ko00330]
Ferroptosis [PATH:ko04216]
RNA degradation [PATH:ko03018]
Thyroid hormone signaling pathway [PATH:ko04919]
Glycosaminoglycan degradation [PATH:ko00531]
AMPK signaling pathway [PATH:ko04152]
Pentose and glucuronate interconversions [PATH:ko00040]
Biofilm formation - Escherichia coli [PATH:ko02026]
Peroxisome [PATH:ko04146]
Tropane
piperidine and pyridine alkaloid biosynthesis [PATH:ko00960]
Nicotinate and nicotinamide metabolism [PATH:ko00760]
Necroptosis [PATH:ko04217]
Plant-pathogen interaction [PATH:ko04626]
Biosynthesis of ansamycins [PATH:ko01051]
Lysosome [PATH:ko04142]
Phenylpropanoid biosynthesis [PATH:ko00940]
Biofilm formation - Pseudomonas aeruginosa [PATH:ko02025]
Protein kinases [BR:ko01001]
Riboflavin metabolism [PATH:ko00740]
Phenylalanine metabolism [PATH:ko00360]
Novobiocin biosynthesis [PATH:ko00401]
Arginine biosynthesis [PATH:ko00220]
Protein phosphatases and associated proteins [BR:ko01009]
Nitrogen metabolism [PATH:ko00910]
Energy metabolism
C5-Branched dibasic acid metabolism [PATH:ko00660]
Carbon fixation in photosynthetic organisms [PATH:ko00710]
Pantothenate and CoA biosynthesis [PATH:ko00770]
Structural proteins
Carbon fixation pathways in prokaryotes [PATH:ko00720]
Citrate cycle (TCA cycle) [PATH:ko00020]
Toll and Imd signaling pathway [PATH:ko04624]
Enzymes with EC numbers
Lipopolysaccharide biosynthesis [PATH:ko00540]
Oxidative phosphorylation [PATH:ko00190]
Two-component system [BR:ko02022]
Glyoxylate and dicarboxylate metabolism [PATH:ko00630]
leucine and isoleucine biosynthesis [PATH:ko00290]
Transcription machinery [BR:ko03021]
Valine
Histidine metabolism [PATH:ko00340]
Bacterial chemotaxis [PATH:ko02030]
Flagellar assembly [PATH:ko02040]
Porphyrin and chlorophyll metabolism [PATH:ko00860]
Cell growth
Phenylalanine
tyrosine and tryptophan biosynthesis [PATH:ko00400]
Bacterial motility proteins [BR:ko02035]
-4 -3 -2 -1 0 1 2 3 4
LDA SCORE (log 10)

Figure S1 LEfSe analysis revealed 139 different functional categories between control and depressed mice.

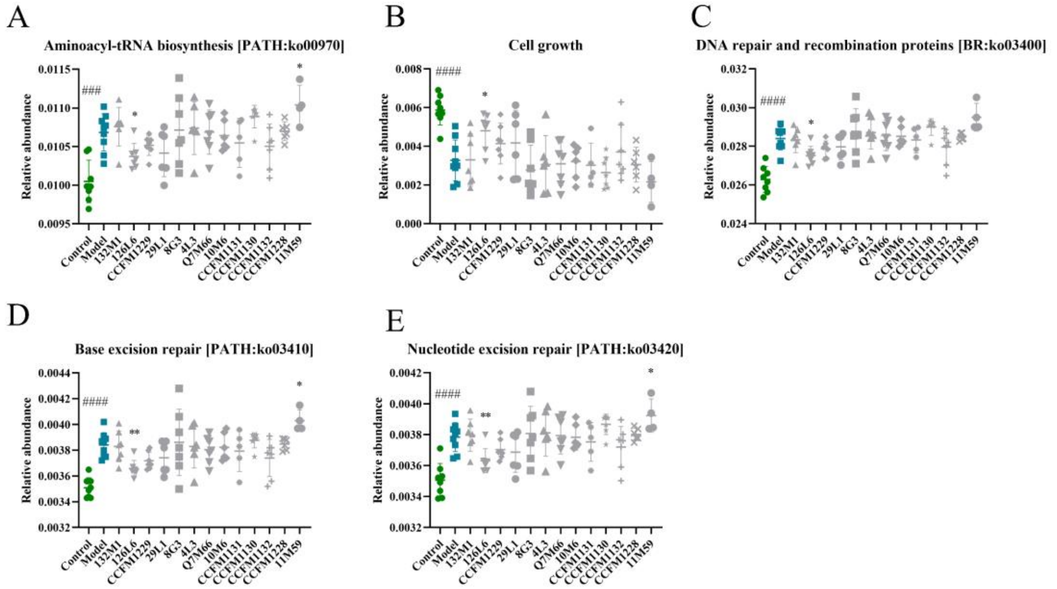

Figure S2 KEGG function prediction of 16S rRNA by PICRUSt2. (A) Aminoacyl-tRNA biosynthesis. (B) Cell growth. (C) DNA repair and recombination proteins. (D) Base excision repair. (E) Nucleotide excision repair. **P*<0.05; ***P*<0.01; ****P*<0.001 comparing with depression group.

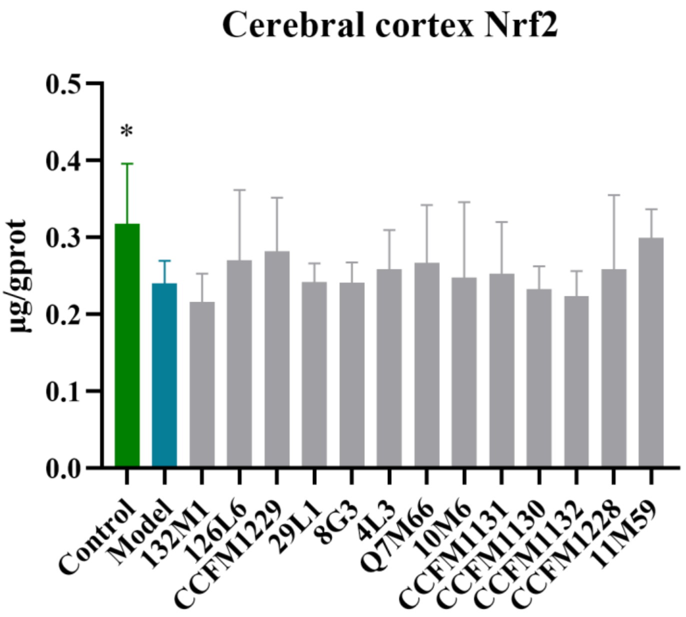

Figure S3 Nrf2 level in mouse cerebral cortex. **P*<0.05.
